# Identification of a novel *MTAP*-*RAF1* fusion in a soft tissue sarcoma

**DOI:** 10.1186/s13000-018-0759-z

**Published:** 2018-10-12

**Authors:** J. Kevin Hicks, Evita Henderson-Jackson, Julia Duggan, David M. Joyce, Andrew S. Brohl

**Affiliations:** 1 DeBartolo Family Personalized Medicine Institute, Department of Individualized Cancer Management, H. Lee Moffitt Cancer Center and Research Institute, Tampa, Florida USA; 2 Department of Anatomic Pathology, H. Lee Moffitt Cancer Center and Research Institute, Tampa, Florida USA; 3 Foundation Medicine, Inc., Cambridge, MA USA; 4 Sarcoma Department, H. Lee Moffitt Cancer Center and Research Institute, 12902 Magnolia Drive, FOB1, Tampa, Florida 33612 USA; 5 Chemical Biology and Molecular Medicine Program, H. Lee Moffitt Cancer Center and Research Institute, Tampa, Florida, USA

**Keywords:** Soft tissue sarcoma, *RAF1*, *MTAP*, Fusion, Next generation sequencing, Molecular diagnostics

## Abstract

**Background:**

RAF family activating fusions have been described as a potentially targetable molecular finding in a subset of soft tissue sarcomas. To further expand upon the landscape of this genetic feature, we describe a novel *MTAP-RAF1* activating fusion identified in a S100 positive soft tissue sarcoma.

**Case presentation:**

A 51 year old man underwent excision of a soft tissue mass in his foot. Pathology revealed a spindle cell neoplasm with S100 positivity, ultimately classified as a soft tissue sarcoma, not otherwise specified. Comprehensive molecular profiling was performed to help establish the diagnosis and revealed a novel *MTAP-RAF1* fusion that includes the tyrosine kinase domain of *RAF1*.

**Conclusions:**

Our report adds to the spectrum of fusion-driven RAF activation observed in soft tissue sarcomas and lends additional evidence that RAF activation plays an important role in some soft tissue sarcomas. Identification of novel fusions involving the MAPK/ERK pathway in sarcomas may provide new avenues for precision medicine strategies involving targeted kinase inhibitors.

## Background

Recently, recurrent *BRAF* gene fusions have been identified in several subgroups of soft tissue sarcomas [1, 2]. To further expand upon the landscape of activating RAF family fusions identified in sarcomas, we report a case of soft tissue sarcoma harboring a novel *MTAP-RAF1* fusion. The case was diagnostically challenging as attempts to classify towards a sarcoma subtype were unsuccessful. In addition to sarcoma subtypes, the diagnoses of a spindle cell melanoma variant or clear cell sarcoma were considered given S100 positivity and the biphasic nature of the tumor. In an attempt to aid in the pathological classification of the tumor, comprehensive molecular profiling was performed and interestingly harbored a novel *MTAP-RAF1* fusion. To our knowledge, this fusion partnering has not previously been described in the literature to date. Given the potentially high-impact nature and novelty of this finding, we report the clinicopathological details of this case to add to the spectrum of RAF family driven soft tissue sarcomas.

## Case presentation

**Fig. 1:**
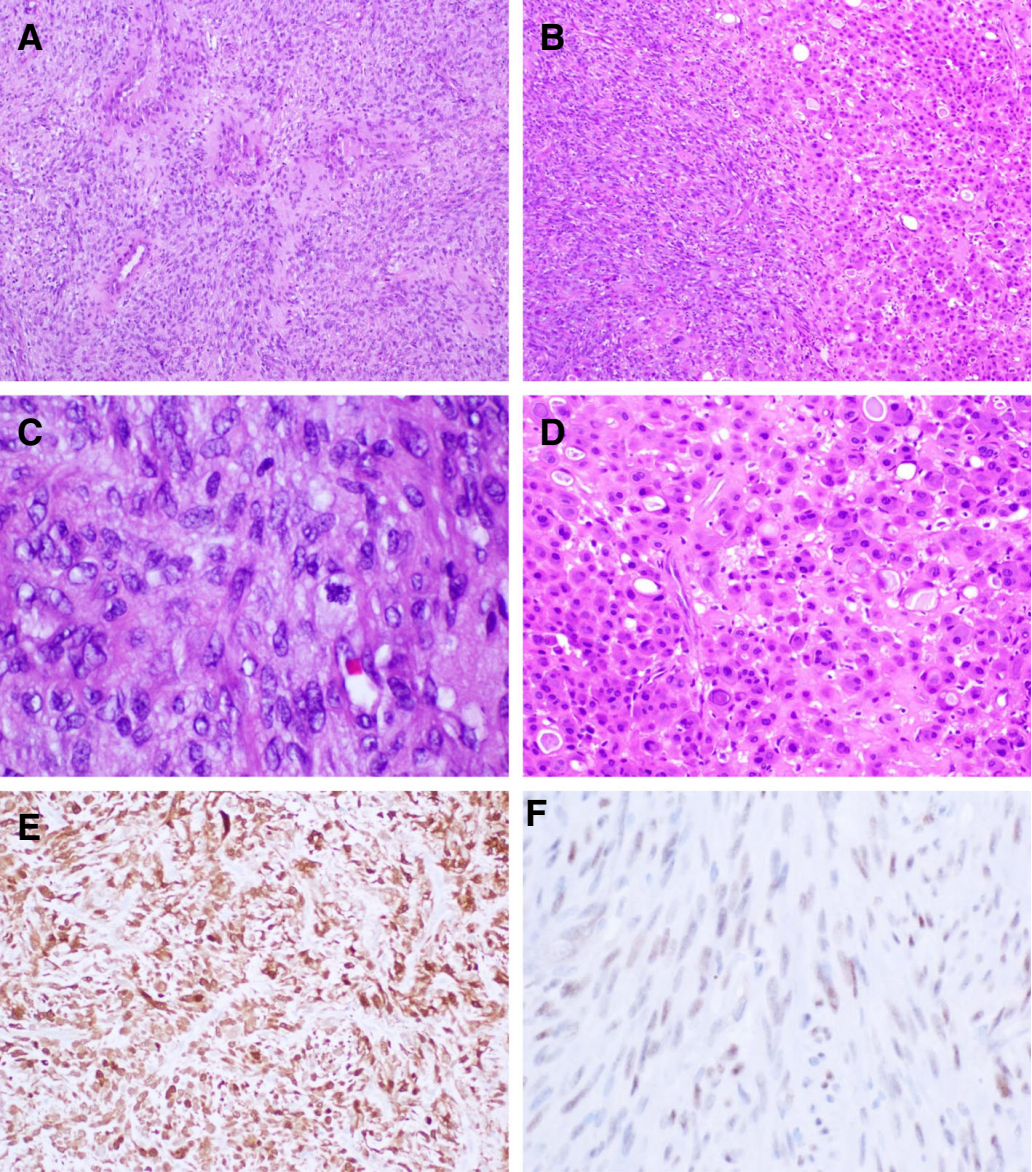
Histological features of the sarcoma. The tumor is composed of (**a**) spindle-shaped cells focally and (**b**) arranged in intersecting fascicles with epithelioid islands. **c** The spindle cells have variable shaped vesicular nuclei with occasional mitoses, (**d**) whereas the epithelioid cells have round to ovoid nuclei and distinct cytoplasmic borders. **e** Tumor cells were positive for S100. **f** Tumor cells show weak nuclear positivity for TFE3

A 51-year-old male presented with a 3 year history of a mass in his right foot with recent enlargement associated with pain. Imaging revealed a soft tissue mass in the plantar arch of the right foot anterior to the calcaneus deep to the fascia. Staging imaging showed no evidence of metastatic disease. The mass was excised. Histologic sections revealed a cellular tumor with scattered ectatic and hyalinized vessels composed of spindle-shaped cells (Fig. 1a) focally arranged in intersecting fascicles admixed with islands of epithelioid cells (Fig. 1b). The spindle cells have vesicular nuclei of variable shape and contour and indistinct cytoplasmic borders with occasional mitoses (Fig. 1c). The epithelioid cells have round to ovoid nuclei with occasional bi-nucleation, and ample eosinophilic cytoplasm, some with focal vacuolation, with distinct cytoplasmic borders (Fig. 1d). No significant necrosis was observed. Immunohistochemical analysis was performed and showed tumor cells positive for S100 (Fig. 1e), specifically the spindle cells were strongly positive while the epithelioid cells were weakly positive. Tumor cells were also positive for TFE3 and vimentin. Immunostains were negative for desmin, MSA, AE1/3, A103, SOX10, MelanA, HMB45, MITF, Tyrosinase and BRAF.

S100 positive, SOX10 negative spindle cell malignancy is a broad pathologic differential. This pattern is observed commonly in a number sarcoma subtypes including synovial sarcoma, Ewing sarcoma, rhabdomyosarcoma, and extraskeletal myxoid chondrosarcoma [3]. S100 positivity is also common in ossifying fibromyxoid tumors, though is typically negative in the malignant cases [4]. SOX10 negative melanoma, clear cell sarcoma, and malignant peripheral nerve sheath tumor (MPNST) are also diagnostic possibilities. We therefore performed additional molecular profiling studies. There was retained nuclear staining of INI-1 and H3K27m3 by immunohistochemistry. Molecular testing performed and interpreted at University of Nebraska, Omaha NE reported the tumor as negative for fusion of the *EWSR1* (22q12) and *ATF1* (12q13) loci, negative for rearrangement of the *TFE3* (Xp11) locus, and negative for fusion of the *EWSR1* (22q12) and *CREB1* (2q33.3) loci. Additionally, no *SS18*/*SSX1* or *SS18*/*SSX2* fusion transcript was detected by RT-DNA amplification. Given the lack of a more specific molecular finding, the malignant S100-positive tumor was therefore favored to be a soft tissue sarcoma that could not be further subtyped, though could not rule out either a spindle cell melanoma or a fusion-negative clear cell sarcoma.

**Fig. 2:**
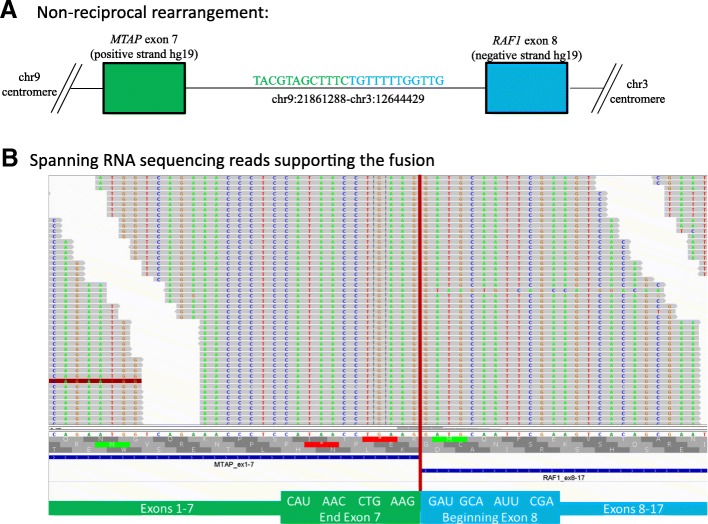
**a** Molecular interrogation found a non-reciprocal gene rearrangement involving *MTAP* on chromosome 9 and *RAF1* on chromosome 3. **b** Supporting RNA sequencing reads spanning the transcriptional breakpoint of the resultant fusion. Sequencing data is visualized in the integrated genomics viewer

Given the uncertainty in diagnosis, reference laboratory molecular tumor profiling was performed to identify additional molecular alterations and to potentially aid in patient management. Hybrid capture-based comprehensive next-generation sequencing (Foundation Medicine Inc., Cambridge, MA) revealed a fusion between *MTAP* exons 1–7 and *RAF1* exons 8–17 (Fig. 2). Presence of the fusion was supported by both DNA evidence (150 supporting read pairs) and RNA evidence (437 supporting read pairs). The fusion product disrupts MTAP (S-methyl-5′-thioadenosine phosphorylase) activity but conserves the RAF1 tyrosine kinase domain with loss of the autoinhibitory N-terminal domain [5]. Loss of MTAP, which functions as a tumor suppressor and is vital for methionine salvage, has been observed in a spectrum of cancers including melanoma and sarcoma [6, 7]. Next-generation sequencing (NGS) also reported a low tumor mutation burden of two mutations per megabase and lack of an ultraviolet (UV)-signature as measured by C → T mutations at dipyrimidine sites. Taken in whole, the clinicopathologic and genomic feature of the case including location and size of the mass (deep soft tissue without cutaneous involvement), immunohistochemistry findings, low mutation burden, and lack of a UV-signature ruled against the possibility of this being a melanoma variant. Therefore a fusion-positive soft tissue sarcoma, not otherwise specified, was the final diagnosis.

## Discussion and conclusions

To our knowledge, we report the first observation of an oncogenic *MTAP-RAF1* fusion. RAF1 is part of the MAPK/ERK pathway, with the fusion product predicted to upregulate signaling. *MTAP* has rarely been reported as a partner gene in cancers, typically in relation to intrachromosomal deletion events of the nearby CDKN2A locus [8]. As a known tumor suppressor, loss of MTAP activity due to a fusion event may also contribute to oncogenesis [9]. Recurrent activating *BRAF* fusions have been reported in several soft tissue sarcoma subtypes [1, 2]. Additionally, *BRAF* activating mutations have been identified in clear cell sarcomas with observed response to vemurafenib [10, 11]. Our report therefore adds to the spectrum of fusion-driven RAF activation observed in soft tissue sarcomas and lends additional evidence that RAF activation plays an important role in some sarcomas. Though systemic therapy was not indicated in the described case, identification of novel fusions involving the MAPK/ERK pathway in soft tissue sarcomas may provide new avenues for precision medicine strategies involving targeted kinase inhibitors.

## Abbreviations

MTAP: S-methyl-5′-thioadenosine phosphorylase
NGS: Next generation sequencing
UV: Ultraviolet
